# Effect of Dentin Pretreatment With Dimethyl Sulfoxide Solution on Interfacial Fracture Toughness of Composite Resin to Wet and Dry Dentin

**DOI:** 10.1155/ijod/5306722

**Published:** 2025-12-02

**Authors:** Fatemeh Molaei, Mehrsima Ghavami-Lahiji, Seyedeh Maryam Tavangar

**Affiliations:** ^1^Dental Sciences Research Center, School of Dentistry, Guilan University of Medical Sciences, Rasht, Iran; ^2^Department of Restorative Dentistry, Dental Sciences Research Center, School of Dentistry, Guilan University of Medical Sciences, Rasht, Iran

**Keywords:** composite resins, dental bonding, dentin, dimethyl sulfoxide

## Abstract

**Objectives:**

This study aimed to investigate the effect of dentin pretreatment with dimethyl sulfoxide (DMSO) on the interfacial fracture toughness (IFT) of composite resin bonded to dentin under varying collagen hydration conditions, addressing conflicting findings in existing literature.

**Methods and Materials:**

A total of 84 dentin samples were randomly assigned to six groups based on the following variables: initial collagen hydration condition (wet or dry), dentin pretreatment (DMSO/ethanol or control), and collagen moisture condition prior to hybridization as follows: (1) Wet dentin control (no pretreatment, blot-dried), (2) Wet dentin + DMSO/EtOH pretreatment (blot-dried), (3) Wet dentin + DMSO/EtOH pretreatment (air-dried), (4) Dry dentin control (no pretreatment, air-dried), (5) Dry dentin + DMSO/EtOH pretreatment (blot-dried), and (6) Dry dentin + DMSO/EtOH pretreatment (air-dried). A two-step etch-and-rinse (E&R) bonding technique was utilized. After bonding, samples were stored in distilled water for 24 h and subjected to 3000 thermocycling cycles. IFT was measured, and fracture patterns were analyzed using stereomicroscopy and scanning electron microscopy (SEM). Statistical analysis employed ANOVA at a significance level of 0.05.

**Results:**

The average IFT revealed significant differences between initial dentin conditions (*p*  < 0.001), with higher values in the wet condition compared to dry. Both initial dentin condition (*p*  < 0.001) and DMSO pretreatment (*p* = 0.010) significantly influenced IFT, while their interaction effect was not significant (*p* = 0.937). Notably, the Dry (Control) group exhibited lower IFT compared to the Dry-DMSO/EtOH-Blot group, which showed higher average IFT (*p* = 0.032). Adhesive failure was the predominant mode across all groups, with no significant differences in fracture patterns.

**Conclusions:**

The DMSO/EtOH solution, used as a dentin pretreatment, positively affected the IFT of composite resin, particularly for wet dentin surfaces.

## 1. Introduction

The introduction of adhesive materials in dental science has drastically changed the way tooth cavities are prepared and restored. Dental adhesives are highly valued in the field, and current studies aim to enhance their mechanical, biological, and durability aspects [1–5]. Despite advancements in bonding to enamel, bonding to dentin using resin remains a challenge due to structural differences [1, 5–7]. Enamel is primarily made up of hydroxyapatite [8–12], while dentin is a dense tissue composed of hydroxyapatite crystals, organic matter, and water, mainly collagen. The tubular structure of dentin and its fluid movement create bonding challenges. Achieving satisfactory bond strength to dentin remains challenging due to its complex structure, including its tubular nature, fluid dynamics, and the susceptibility of the hybrid layer to degradation by matrix metalloproteinases (MMPs) [8–10, 13–16].

Dental adhesives are categorized into two groups: self-etching and etch-and-rinse (E&R), depending on whether they require a separate surface etching step [17, 18]. The E&R group commonly employs the wet bonding method, which is a standard procedure [7, 19]. However, this method is highly sensitive to potential issues like over-drying and over-wetting, each of which can significantly affect the adhesive's performance [7, 16, 20–22]. Excessive residual water can weaken the resin–dentin bond by creating a weak hybrid layer, which reduces long-term durability [5, 22, 23]. This excess water leads to several challenges, including the separation of adhesive components during hybridization, increased hydrolysis of polymers with ester bonds over time, and the activation and release of collagen-hydrolyzing enzymes, such as MMPs and cathepsins from the host [5, 22–25]. Hydrolytic instability at the resin-dentin interface is an unavoidable issue in wet bonding. To improve dentinal bonding, hybrid layers with fewer pores and reduced water content and affinity are needed [22, 23, 25]. However, accurately controlling humidity with existing instruments is often impractical, making it difficult to achieve an effective bond in wet conditions [7, 23].

One suggested method to improve wet bonding is ethanol wet bonding (EWB). EWB involves gradually replacing water in demineralized dentin with resin monomers using ethanol [26–28]. This process allows the hydrophobic resin to penetrate the dentin hybrid layer, enhancing wet bonding and reducing problems associated with excess moisture. However, ethanol cannot entirely remove water due to the strong bond between collagen fibers and water [5, 23, 27, 28].

The classic dry bonding technique is another method to control moisture on dentin surfaces. It involves using air to dry the dentin after etching and rinsing. However, based on previous studies, this method aims to remove remaining water, achieving less-than-optimal dentin–resin bonding [7, 22, 23]. The main challenge is that resin-solvent mixtures cannot effectively restore dried and collapsed collagen, limiting the effectiveness of dry bonding. Excessive drying causes the collapse of water between collagen molecules, reducing the gaps that normally allow resin penetration. This collapse compromises the micromechanical bond, leading to reduced long–term bond durability and strength [7, 15, 16, 22, 23, 25].

Dimethyl sulfoxide (DMSO) is a multifunctional solvent known for its capability to dissolve both polar and nonpolar substances. In dentistry, it is soluble in a variety of organic solvents, water, and common resin monomers [6, 23, 29, 30]. Its small molecular size, dipolar aprotic nature, and ability to interact with collagen fibers make DMSO a promising candidate for improving dentin–resin bonding [5, 29, 30].

Research has shown that DMSO can enhance both immediate and long-term resin–dentin bond strength [5, 6]. DMSO's unique features include modifying demineralized type I collagen by forming hydrogen bonds with proteins, increasing collagen fibril spacing, and improving resin penetration. It also helps to separate residual water from dentin surfaces, increasing the wettability and infiltration of monomers. Additionally, DMSO reduces the activity of hydrolytic enzymes, like MMP 9 and MMP 2, protecting collagen from degradation. DMSO's role in radical polymerization within solvent-monomer mixtures further enhances its effectiveness in resin–dentin bonding [5, 6, 19, 25, 29, 30].

The inclusion of DMSO as a novel solvent in the dry bonding method has shown promising outcomes. Recent studies demonstrated that DMSO surface modification under dry bonding conditions improves the reliablity, and consistency of bonding mitigating the disruption caused by residual water layers surrounding collagen [5, 7, 23, 30].

Interfacial fracture toughness (IFT) measures the resistance of adhesive interfaces to crack initiation and propagation, providing a more comprehensive assessment of bonding effectiveness than conventional bond strength tests. Unlike shear bond strength tests that apply simplified load until failure, IFT accounts for both the interfacial bond strength and inherent defects present at the interface, reflecting clinical failure mechanisms more accurately [31–33]. Specifically, IFT evaluates resistance under mixed-mode stresses, analogous to those occurring during mastication and thermal cycling in the oral environment, thus assessing bond durability and longevity more effectively.

Previous studies using three-step E&R and universal adhesive systems have examined DMSO's effects on bond strength [7, 23, 34]; however, data on two-step E&R adhesives remain limited. Moreover, fracture toughness testing has not yet been applied to assess DMSO-biomodification techniques. Given that conventional bond strength tests can be influenced by uncontrolled defect distribution at the interface, IFT provides a more reliable measure of adhesive resistance to failure.

Therefore, this study aimed to evaluate the effects of DMSO mixture pretreatments on the IFT of composite resin bonded to dentin under varying collagen hydration conditions, using two-step E&R adhesive systems. The study examined pretreatments applied to both wet and severely air-dried dentin, followed by blot or air drying. The null hypotheses were that, under each initial moisture condition (wet or dry), DMSO pretreatment followed by blot or air drying would have no effect on the bonding efficacy of composite resin to dentin.

## 2. Materials and Methods

Human third molars that were extracted during routine dental procedures were utilized for this study. The use of extracted third molars was approved by the Ethical Committee of Guilan University of Medical Sciences under IR.GUMS.REC.1402.017 number. The teeth were initially decontaminated in 0.5% chloramine T at 4°C for 7 days, and subsequently transferred to distilled water until beginning the experiment.

To determine the sample size, the formula appropriate for two-way ANOVA analysis was used with the G^*⁣*^*∗*^^Power 3.1 software. For this purpose, the effect size was set at 0.35 (considered a medium effect size according to Cohen's classification), along with a statistical power of 0.80 and a significance level of 0.05. The number of groups, based on the number of factors under investigation, was set to 6, and the degrees of freedom were specified as 2. As a result, the sample size for each group was calculated to be 14.

Consequently, the total sample size was determined to be 84. This was derived by dividing the total of 82 obtained from the software by the number of groups (6), which resulted in approximately 13.67 and was rounded up to 14. Below, the results obtained from G^*⁣*^*∗*^^Power 3.1 software are presented.

**Figure 1:**
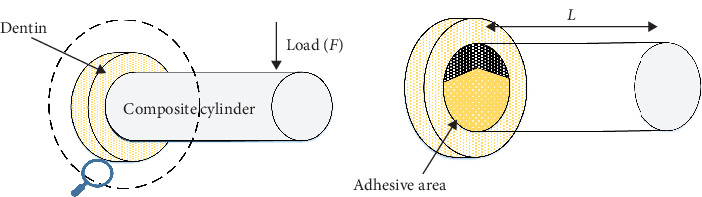
Illustration of bonded samples and loading setup for measuring IFT. A bonding area with a chevron shape beneath the composite tube is shown on the right.

The teeth were then set in acrylic resin cast, and the coronal enamel was removed using a low-speed saw (Buehler Ltd, Lake Bluff, Illinois, USA) with water cooling. The exposed dentin was then ground with wet 400, 600, and 800-grit SiC papers to create a standardized smear layer. Careful inspection was carried out with a stereomicroscope (SZ51; Olympus, Tokyo, Japan) to ensure the absence of enamel. The procedure for assessing the IFT has been previously documented. A bonding area of 4 mm in diameter, featuring a 90° chevron shape, was created using a polytetrafluoroethylene tape produced with a custom-made punch. The tape was burnished to prevent adhesive and composite flashing [31]. In this method, a tape is placed on the dentin surface before bonding to create a standardized notch, as shown in the gray area in the schematic picture (Figure 1).

Eighty-four extracted teeth were randomly allocated into six experimental groups (*n* = 14). These groups were defined based on two factors: the dentin moisture condition (wet or dry) and the type of pretreatment applied (control, DMSO/EtOH followed by blot drying, or air drying).

All dentin surfaces were etched with 37% phosphoric acid (Condac 37%, FGM, Joinville, Santa Catarina, Brazil) for 15 s, followed by water rinsing for 30 s. The surfaces were then subjected to one of two moisture management methods: blot-drying, ensuring no visible moisture remained on the surface (referred to as wet dentin), or air-drying by continuous air blast for 30 s (dry dentin). Control groups did not undergo DMSO pretreatments, adhering to traditional wet- or dry-bonding approaches. Equal volumes of DMSO (Sigma Aldrich, St. Louis, MO, USA) were mixed with ethanol (Ethanol 99.8%, Sigma–Aldrich) to create 50% DMSO (v/v) solutions right before use [6, 7, 25]. The resulting solution was then applied to the etched dentin surfaces of the DMSO/EtOH pretreatment groups for 60 s. Moisture control after applying DMSO/EtOH was also achieved through either blot-drying, ensuring no visible moisture remained on the surface, or air drying by continuous air blast for 30 s.

The specific procedures for each group are detailed below:

1. Wet dentin Control: The bonding area was etched and rinsed, then gently dried by blot-drying to retain moisture without visible wetness. No DMSO/EtOH pretreatment was applied.
2. Wet dentin DMSO/EtOH-Blot dry: Following the same etching, rinsing, and blot-drying procedure as the group 1, the bonding area was treated with DMSO/EtOH for 60 s, then gently blot-dried to remove visible moisture.
3. Wet dentin DMSO/EtOH-Air dry: After etching, rinsing, and blot-drying as in group 1, the bonding area received the DMSO/EtOH treatment for 60 s, followed by air drying with a continuous air blast for 30 s.
4. Dry dentin Control: The bonding area was etched and rinsed, then air-dried with a continuous air blast for 30 s. No DMSO/EtOH pretreatment was applied.
5. Dry dentin DMSO/EtOH-Blot dry: Following etching, rinsing, and air drying as in group 4, the bonding area was treated with DMSO/EtOH for 60 s, then blot-dried.
6. Dry dentin DMSO/EtOH-Air dry: After etching, rinsing, and air drying as in group 4, the bonding area received DMSO/EtOH treatment for 60 s and was subsequently air-dried with a continuous air blast for 30 s.

A water-based two-step E&R adhesive (Ambar APS; FGM, Joinville, Santa Catarina, Brazil) was used according to the manufacturer's instructions to all groups (Table 1). The composite restorations were incrementally built up by 2 mm on the bonded surfaces with plastic cylinders with inner diameters of 4 mm and heights of 10 mm (Candohydraulic, Tehran, Iran). Each layer was individually light cured for 20 s using an LED curing unit (Blue dent, LED Smart, SC, Brazil) with a light intensity of 1200 mW/cm^2^. Sample preparations were carefully performed under magnification using a dental loupe (AMTECH TTL, Wenzhou, China). Following this, the samples were immersed in distilled water at 37°C for 24 h and afterwards underwent 3000 cycles of thermocycling, involving alternating water baths between 5 and 55°C, with a dwell time of 20 s [35–38].

For the evaluation of IFT, a universal testing machine (STM-20; SANTAM Engineering and Design Co., Tehran, Iran) was employed. The specimens have been subjected to compressive forces at a crosshead speed of 1 mm per minute applied nearly 9 mm from the bonded interface [31]. The following formula was used to calculate the IFT values of each specimen:

  $$\begin{array}{c} G_{\text{IC}}=\frac{104\cdot F^{2}L^{3}}{{\text{ED}}^{6}}. \end{array}$$

The critical plane strain energy release rate (*G*_IC_) is measured in J/m^2^, the load at which breakage occurs is denoted by *F* (N), *L* represents the distance to the loading point in millimeters (mm), *D* is the diameter of the resin cylinder in mm, and *E* stands for the elastic modulus of the resin, which is 15.68 GPa for Opallis composite resin [39]. All fractured patterns were examined using a stereomicroscope (SZ51; Olympus, Tokyo, Japan) with a 40x magnification. Two samples from each group were chosen for further evaluation using scanning electron microscopy (SEM) (Vega Tescan, Brno, Czech Republic) at ×20, ×50, and ×200 magnification.

Fracture modes were categorized into adhesive failure (failure at the interface between the resin and dentin), cohesive failure (failure occurring only within the dentin or resin composite), and mixed failure (failure at the resin/dentin interface with cohesive failure of the adjacent substrates). To reduce observer bias, all specimens were randomly coded, and the operators performing the IFT and failure mode evaluations under a stereomicroscope and SEM were blinded to the group assignments.

The normality of the data distribution was assessed using the Shapiro–Wilk test, and the homogeneity of variances was evaluated with Levene's test. For inter-group comparisons across different pretreatment groups under various initial dentin moisture conditions, one-way ANOVA was performed, followed by Tukey's post hoc test for pairwise analyses. In addition, two-way ANOVA was used to examine the interaction effects between the “initial dentin moisture” factor and the “dentin pretreatment” factor. To explore intra-group pairwise differences within each pretreatment group under different initial moisture conditions, independent samples *t*-tests were conducted. All statistical analyses were performed using IBM SPSS Statistics, version 28 (IBM Corp., Armonk, NY, USA), with a significance level of *α* = 0.05 for all tests.

## 3. Results

The effect of dentin pretreatment protocols (control, DMSO–blot-drying, DMSO–air-drying) on the IFT of composite resin to dentin was significant (*p* = 0.027). The blot-drying group exhibited a higher mean IFT compared with both the control (*p* = 0.026) and air-drying (*p* = 0.041) groups.

The interaction between initial dentin moisture and dentin pretreatment was not statistically significant (*p* = 0.937). However, each factor independently influenced IFT, with a significant effect observed for both initial dentin moisture (*p*  < 0.001) and dentin pretreatment (*p* = 0.010).

Table 2presents the mean IFT (J/m^2^) for all groups under wet- and dry-dentin conditions. Under wet-dentin conditions, DMSO–blot-drying produced higher IFT than the control, approaching significance (*p* = 0.052). Under dry-dentin conditions, DMSO–blot-drying significantly increased IFT compared with the control (*p* = 0.032).

Within each group, IFT values under wet conditions were significantly higher than under dry conditions for the control (*p* = 0.009), DMSO–blot-drying (*p* = 0.040), and DMSO–air-drying groups (*p* = 0.008). Between wet-dentin groups, no significant difference was observed between the two DMSO pretreatments (*p* = 0.101). Between dry-dentin groups, no significant difference was observed between the two DMSO pretreatments (*p* = 0.080).

**Figure 2:**
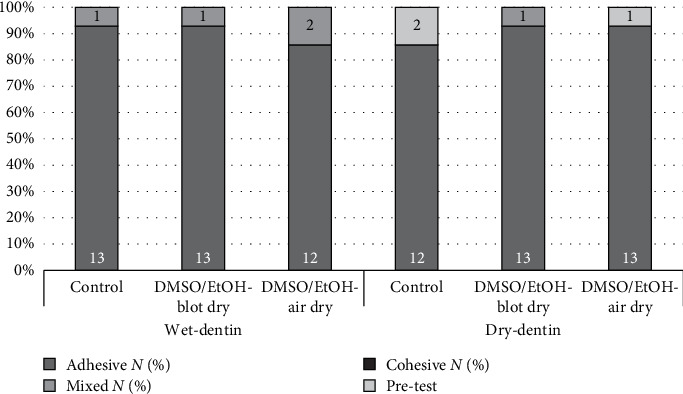
Distribution of fracture patterns observed for tested specimens after IFT test.

Table 3 summarizes the failure mode distribution. The majority of failures in all groups were adhesive, followed by mixed failures. Notably, no specimen exhibited total cohesive failure within the adjacent substrates. The distribution of failure modes was unaffected by surface pretreatment (*p* = 0.836) or by the interaction between dentin moisture and pretreatment (*p* = 0.724; Figure 2).

## 4. Discussion

That study examined the effect of dentin pretreatment with a DMSO/ethanol solution on the IFT of composite resin bonded to dentin under different moisture conditions. The null hypotheses were rejected because DMSO pretreatment significantly improved IFT values, especially in wet dentin. The combination of DMSO pretreatment and blot-dry protocols also mitigated some negative effects of air drying on dry dentin bonding.

Water has been shown to have opposing effects on the resin–dentin bonding. While excessive moisture may limit the diffusion of hydrophobic monomers to the depth of the hybrid layer, which could be harmful to polymer formation and impair the degree of conversion of resin matrix, a lack of moisture limits interfibrillar gaps, which compromises the diffusion of adhesive monomers [22, 23]. Despite the promising results of the EWB method in enhancing resin–dentin bond durability, it has a time-consuming and technique-sensitive protocol when used for dentin bonding. In addition, full substitution of water with ethanol cannot be performed in a clinical setting owing to factors, such as the continuous presence of physiological dentin fluid in the dental pulp [40–42].

In E&R adhesives, controlling the surface moisture of dentin until ideal is almost impossible in a clinical setting [7, 43]. Air-drying, which is simpler than blot-drying, is one method of moisture control. However, the most serious issue with this moisture control method is the collapse of collagen, and the incapacity of the resin–solvent mixture to re-expand the collapsed dry collagen impairs the performance of dry bonding [7].

DMSO is a polar aprotic solvent with very low vapor pressure. A polar aprotic solvent is a solvent that lacks acidic protons. Such solvents do not contain hydroxyl and amine groups. Unlike protic solvents, they do not act as proton donors in hydrogen bonds, although they can be proton acceptors. Polar aprotic solvents are particularly important because of their ability to dissolve salts and both polar and nonpolar compounds [5, 44, 45]. DMSO is a highly effective solvent that can completely dissolve most monomers in dental adhesives. It can penetrate biological surfaces and interact with collagen fibers due to its small size, dipolar aprotic nature, and ability to dissolve polar and nonpolar compounds. In addition to producing higher initial bond strength, recent investigations on DMSO have demonstrated encouraging outcomes, including prolonged bond strength and surfaces with less residual water when applied utilizing the dry bonding approach [5, 44, 45]. DMSO enhances the wettability of demineralized dentin by dissociating the remaining water from the dentin surface, facilitating monomer penetration into demineralized dentin. Moreover, DMSO can limit the action of endogenous hydrolytic enzymes, such as MMP 9 and MMP 2, thereby preventing collagen degradation. It also reduces the technical sensitivity of E&R dentin bonding agents, even after over-drying the dentin. DMSO can improve the different aspects of resin–dentin bonding by facilitating the radical polymerization process in solvent-monomer mixtures [5, 19, 25, 29, 30].

It seems that whereas DMSO surface modification has no effect on polymerization, it can produce significant increases in collagen stiffness that are required to keep the material from collapsing following an air-drying process. DMSO has a low vapor pressure that stops it from completely evaporating, in contrast to ethanol, which readily evaporates from collagen after air drying. Even after extended air drying, the residual DMSO molecules can contribute to the preservation of interfilament gaps that permit the proper diffusion of monomers [7].

The positive impact of DMSO on dentin bonding showed in the present study agrees with prior research reporting that DMSO expands collagen interfibrillar spacing, improves dentin wettability, and inhibits endogenous enzymatic degradation, thereby facilitating deeper monomer infiltration and more stable hybrid layers. Particularly, Stape et al. [25] demonstrated that 50 vol% DMSO in water or ethanol improves bond strength and hybrid layer integrity under wet and dry bonding protocols. They further showed that incorporating 10 wt% DMSO into the adhesive resin (Scotchbond Multi-Purpose primer) enhances resin–dentin interface stability by preventing collagen collapse

Regarding application time, there is variability in the literature between 30 and 60 s of DMSO pretreatment. Al-Ani et al. [46] applied 30-s DMSO pretreatment at concentrations from 0.001% to 20%, showing improved bond durability in 1% and 5%. Stape et al. [7, 23, 25] similarly used 30–60-s applications, reporting beneficial effects. However, de Mello et al. [34] found that 60-s DMSO pretreatment did not improve bonding or interface micromorphology of universal adhesive (Scotchbond Universal). Contrastingly, Cardenas et al. [47] reported improved bonding performance with 60-s pretreatment in two universal adhesives on both sound and eroded dentin. These discrepancies may arise from differences in adhesive systems, solvent mixtures, and testing protocols.

DMSO concentration is another determinant of bonding outcomes. Literature reports concentrations ranging from very low (~0.001%–0.004%) to high (~50%) diluted in water or ethanol. Low concentrations have limited effects, as seen in 0.001% in Al-Ani et al. [46] and 0.004% in Sharafeddin et al. [48], while high concentrations (close to 50%) better maintain collagen hydration and expand interfibrillar spaces. concentrations above 10% sometimes correlate with adverse effects due to potential over-wetting or polymerization interference. The 50 vol% DMSO in ethanol solution used in this study achieves a favorable balance by capitalizing on DMSO's solvent properties for biomodification, while minimizing potential negative outcomes.

In recent studies, the focus has evolved to using the dry bonding technique to eliminate excess water while combining with pretreatment solutions containing DMSO/H_2_O or DMSO/EtOH to improve collapsed collagens [7, 22, 25]. It has been reported that DMSO-pretreated groups exhibit improved wettability, as well as enhanced initial and aged bond strengths. Additionally, these groups show reduced leakage. DMSO pretreatments not only increase bond strength but also result in hybrid layers with reduced porosity after aging, independent of moisture control method. Moreover, DMSO pretreatment lowers the contact angle between water and dentin collagen, indicating improved wettability and facilitating faster resin spreading [7, 23, 25, 46].

The current study revealed that the IFT values for the DMSO-pretreated groups were higher compared to the corresponding control group. This improvement can be attributed to dentin surface biomodifications in both wet and dry dentin subgroups, consistent with previous research. However, Stape et al. [23] demonstrated similar bond strength improvements when comparing DMSO pretreated wet and dry dentin groups, finding comparable results regardless of dentin moisture. Another study showed that air-drying, whether performed before or after DMSO pretreatment, did not significantly affect bond strength to etched dentin, reporting similar outcomes between wet and dry pretreatment groups under different drying methods [7]. While the current study also observed improvements consistent with these findings, the mean IFT values for initially wet dentin were significantly higher than for initially dry dentin (*p*  < 0.001).

These discrepancies may be explained by differences in adhesive and composite brands, ingredient formulations, bonding efficacy test methods, and the application of thermal cycling to simulate oral circumstances.

The collapse of dentin matrices during dry bonding is an active process involving the quick and spontaneous formation of new hydrogen bonds between neighboring collagen peptides, which reduces interfibrillar gaps. Adhesives cannot re-expand the matrix unless they break these interpeptide hydrogen bonds [49]. Depending on whether its hydrogen bonding Hoy's solubility characteristics surpass 14.8 (J/cm^3^)^½^, which is *σ*H solubility parameters of dry collagen without water, solvents may either totally (water) or partially (ethanol, propanol, and acetone) re-expand collapsed collagen [49]. The higher the *σ*H is, the faster and wider the collagen expansion takes place; however, most dental monomers have *σ*H values lower than 14.8, limiting their ability to re-expand dry demineralized dentin. The DMSO–ethanol mixture used in the present study has a higher *σ*H (16.6 [J/cm^3^]^1/2^), indicating greater potency to disrupt hydrogen bonds and re-expand collagen. Compared to hydrophilic adhesives containing HEMA, this DMSO mixture more effectively restores collagen interfibrillar spacing.

If the collagen matrix is too soft, it cannot resist shrinkage forces during solvent evaporation, leading to matrix collapse and compression of unpolymerized monomers before polymerization. Studies show that matrices infiltrated with ethanol mixtures containing adhesive resin components exhibit higher stiffness and less shrinkage compared to water mixtures. Ethanol's higher *σ*H facilitates partial interpeptide hydrogen bonding, pre-stiffening the matrix and reducing shrinkage upon evaporation. This enables more HEMA retention within the matrix, protecting collagen fibers and reinforcing the hybrid layer [49].

It has been reported that replacing water with ethanol in a surface modification solution could enhance the wettability of hydrophobic bonding resins. Ethanol is more efficient than water as a solvent for high molecular weights methacrylate monomers like Bis-GMA, which justifies the combined use of ethanol and DMSO in this study.

The IFT test was employed to evaluate the material's resistance to crack initiation and propagation at the adhesive interface. This method provides a more accurate assessment of interfacial fracture resistance than conventional bond strength tests by considering both interfacial defects and bond strength, utilizing controlled crack length and location [33]. The modified fracture toughness test effectively measures critical energy release rate (*G*_IC_), with sample geometry playing a crucial role in validity [50, 51].

**Figure 3:**
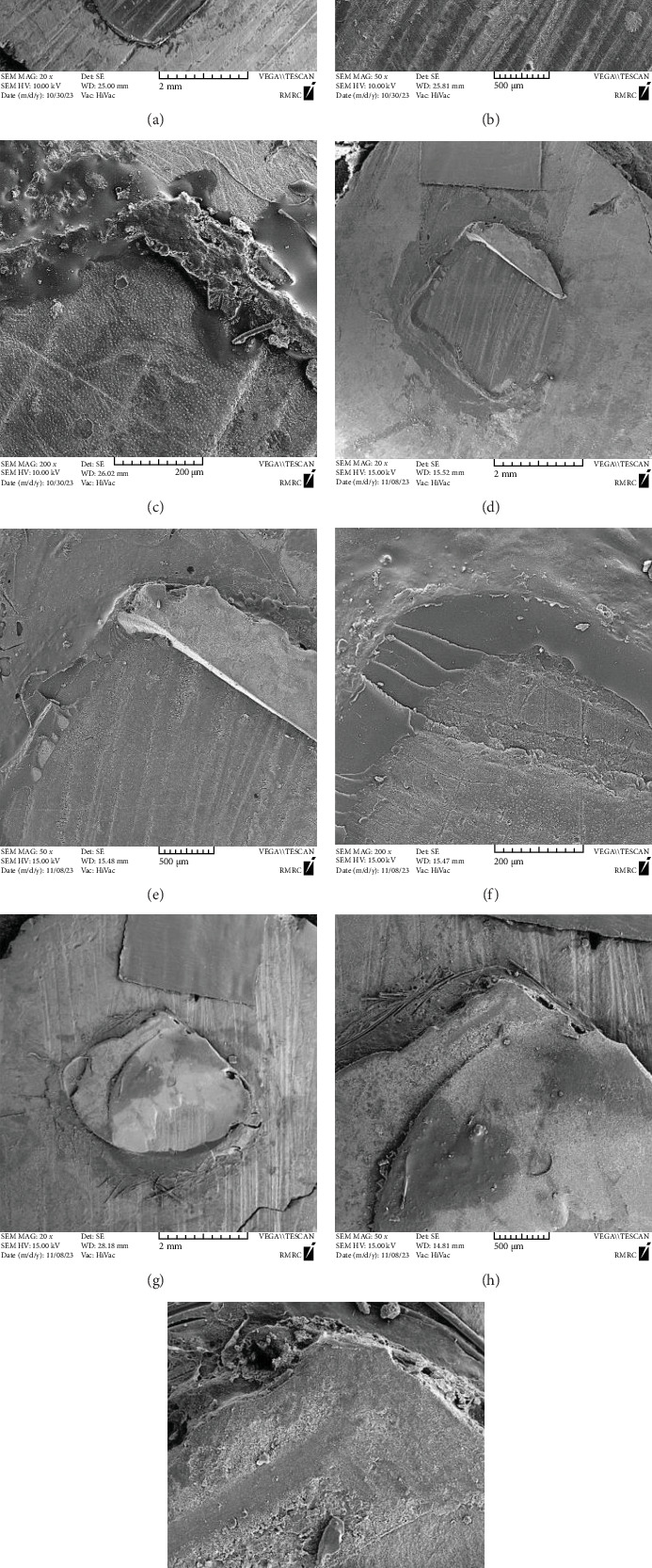
SEM photomicrographs of (a) an adhesive fracture in a specimen, magnification 20x, and (b, c) higher magnifications, (d, e) a mixed interface-resin fracture in a specimen, magnification 20× and 50×, and (f) chevron tip, magnification 200×. Panels (g–i) show a specimen exhibiting predominantly cohesive fracture, with a small proportion of adhesive failure still visible in panel (g).

Adhesive failure was the predominant mode observed, followed by mixed failures, consistent with findings reported by Tantbirojn et al. [51] and Keshvad et al. [31]. In this study, the parts of cohesive fracture areas in resin composite were still adjacent to adhesive failure zones, indicating that the fracture path did not tend to deviate towards the substrate. The presence of mixed failures may be attributed to localized weakening of the substrate during unstable crack growth, which likely explains the failure pattern observed in Figure 3.

This study differed from earlier mini-fracture toughness research conducted by Pongprueksa et al. [33] by showing mixed fracture patterns, possibly due to interface defects, bubbles, or flaws in crack initiation locations. The combination of dentin, adhesive, and composite resin substrates, each with different mechanical properties, can influence failure modes and data variability. While fracture toughness tests are typically suited for brittle materials like ceramics, dentin and adhesives are somewhat brittle, causing variable crack propagation and mixed failure modes.

It should be mentioned that this study is limited to an in vitro setting, serving as a pre-clinical screening, which may not fully represent the complexities of the oral environment. Clinical studies are necessary for further confirmation of these findings. Additionally, only one type of E&R adhesive system and composite resin were investigated. Therefore, the findings may not be directly generalizable to other adhesive systems and composite resins available on the market.

Moreover, the sample preparation and creating a standardized notch in IFT test were complex procedures that were highly dependent on the skill and technique of the operators. This may have introduced some variability in the quality of sample preparation, which could have potentially influenced the results. Future studies should consider standardizing the sample preparation to minimize the impact of operator-dependent factors. Overall, the study provides valuable insights into the use of DMSO/EtOH as a surface modification technique and its impact on the IFT of dentin, particularly in the context of moisture control. These findings may have important implications for the development of more effective adhesive strategies and the optimization of dentin bonding procedures in clinical practice.

## 5. Conclusion

DMSO/ethanol pretreatment significantly enhances IFT of both wet and dry dentin, with a greater effect on moist dentin. No significant difference was found between blot-dry and air-dry protocols after DMSO treatment, indicating both moisture control methods are effective. Importantly, combining blot-dry with DMSO pretreatment partially mitigates the negative impact of drying on dry dentin, supporting its clinical potential to improve adhesive performance.

## Figures and Tables

**Table 1:** Description of materials used.

Material	Composition	Manufacturer
Composite resin: Opallis	Monomer matrix: Bis-GMA, Bis-EMA, TEGDMA, UDMA Fillers: bariun aluminum, silanized silicate, silicon dioxide, camphorquinone, accelerators, stabilizer and pigments Inactive ingredients: silanized bariumaluminium silicate glass, pigments, and silica	FGM Joinville, SC, Brazil
Phosphoric Acid Etching: Gel Condac 37	Phosphoric acid 37%, thickener, pigment, and deionized water	FGM–Condac 37%, Brazil
Adhesive: Ambar APS	MDP, UDMA, methacrylate acidic monomers, methacrylate hydrophilic monomers ethanol, silica nanofiller, photoinitiators, coinitiators, stabilizers	FGM–ambar APS, Brazil

**Table 2:** IFT data (mean ± SD) (J/m^2^) for all groups.

Study group	Wet-dentin	Dry-dentin	The effect of dentin moisture on each group (*p*-Value)^1^
Control	118.78 ± 43.6^Aa^	72.658 ± 38.2^Ab^	0.009
DMSO/EtOH-Blot dry	163.80 ± 70.06^Aa^	114.217 ± 54.3^Ab^	0.04
DMSO/EtOH-Air dry	155.66 ± 55.9^Aa^	99.33 ± 44.03^Ab^	0.008
*p*-Value^2^	0.101	0.080	—

*Note:* Values marked with different lowercase superscript letters in each row are significantly different. Values marked with different uppercase superscript letters in each column are significantly different.

^1^Independent samples test.

^2^ANOVA.

**Table 3:** Mode of failure data for all groups.

Study group	Wet-dentin			Dry-dentin		
Fracture pattern	Adhesive *N* (%)	Mixed *N* (%)	Cohesive *N* (%)	Adhesive *N* (%)	Mixed *N* (%)	Cohesive *N* (%)
Control	13 (92.9)	1 (7.1)	0 (0)	12 (100)	0 (0)	0 (0)
DMSO/EtOH-blot dry	13 (92.9)	1 (7.1)	0 (0)	13 (92.9)	1 (7.1)	0 (0)
DMSO/EtOH-air dry	12 (85.7)	2 (14.3)	0 (0)	13 (100)	0 (0)	0 (0)

## Data Availability

The data that support the findings of this study are available from the corresponding author upon reasonable request.

## References

[1] Perdigão J. (2020). Current Perspectives on Dental Adhesion: (1) Dentin Adhesion–Not There yet. *Japanese Dental Science Review*.

[2] Dionysopoulos D., Gerasimidou O., Papadopoulos C. (2022). Current Modifications of Dental Adhesive Systems for Composite Resin Restorations: A Review in Literature. *Journal of Adhesion Science and Technology*.

[3] Sebold M., André C. B., Sahadi B. O., Breschi L., Giannini M. (2021). Chronological History and Current Advancements of Dental Adhesive Systems Development: A Narrative Review. *Journal of Adhesion Science and Technology*.

[4] Dressano D., Salvador M. V., Oliveira M. T. (2020). Chemistry of Novel and Contemporary Resin-Based Dental Adhesives. *Journal of the Mechanical Behavior of Biomedical Materials*.

[5] Mirzaei K., Ahmadi E., Rafeie N., Abbasi M. (2023). The Effect of Dentin Surface Pretreatment Using Dimethyl Sulfoxide on the Bond Strength of a Universal Bonding Agent to Dentin. *BMC Oral Health*.

[6] Stape T. H. S., Mutluay M. M., Tjäderhane L., Uurasjärvi E., Koistinen A., Tezvergil-Mutluay A. (2021). The Pursuit of Resin-Dentin Bond Durability: Simultaneous Enhancement of Collagen Structure and Polymer Network Formation in Hybrid Layers. *Dental Materials*.

[7] Stape T. H. S., Uctasli M., Cibelik H. S., Tjäderhane L., Tezvergil-Mutluay A. (2021). Dry Bonding to Dentin: Broadening the Moisture Spectrum and Increasing Wettability of Etch-and-Rinse Adhesives. *Dental Materials*.

[8] Gil-Bona A., Bidlack F. B. (2020). Tooth Enamel and Its Dynamic Protein Matrix. *International Journal of Molecular Sciences*.

[9] Beniash E., Stifler C. A., Sun C.-Y. (2019). The Hidden Structure of Human Enamel. *Nature Communications*.

[10] Belmar da Costa M., Delgado A. H., Pinheiro de Melo T., Amorim T., Mano Azul A. (2021). Analysis of Laboratory Adhesion Studies in Eroded Enamel and Dentin: A Scoping Review. *Biomaterial Investigation in Dentistry*.

[11] DeRocher K. A., Smeets P. J., Goodge B. H. (2020). Chemical Gradients in Human Enamel Crystallites. *Nature*.

[12] Zhao H., Liu S., Wei Y. (2022). Multiscale Engineered Artificial Tooth Enamel. *Science*.

[13] Varghese A. S., Prakash V., Mitthra S. (2021). Role Of Different Collagen Cross-Linking Agents Like Proanthocyanidin, Riboflavin and White Tea on the Shear Bond Strength to Dentin-An In-Vitro Study. *NVEO – Natural Volatiles & Essential Oils*.

[14] van den Breemer C., Özcan M., Cune M., Ayres A. A., Van Meerbeek B., Gresnigt M. (2019). Effect of Immediate Dentin Sealing and Surface Conditioning on the Microtensile Bond Strength of Resin-Based Composite to Dentin. *Operative Dentistry*.

[15] Yu F., Luo M., Xu R. (2021). A Novel Dentin Bonding Scheme Based on Extrafibrillar Demineralization Combined With Covalent Adhesion Using a Dry-Bonding Technique. *Bioactive Materials*.

[16] de Alencar M. L., Leite S., de Farias Charamba C. (2020). Bond Strength of Universal Adhesive Applied to Dry and Wet Dentin: One-Year In Vitro Evaluation. *Brazilian Journal of Oral Sciences*.

[17] Gupta A., Tavane P., Gupta P. K. (2017). Evaluation of Microleakage with Total Etch, Self Etch and Universal Adhesive Systems in Class V Restorations: An In Vitro Study. *Journal of Clinical and Diagnostic Research*.

[18] Tran X. V., Tran K. Q. (2021). Microleakage and Characteristics of Resin-Tooth Tissues Interface of a Self-Etch and an Etch-and-Rinse Adhesive Systems. *Restorative Dentistry & Endodontics*.

[19] Luiza Szesz A., Abreu Pereira G. d. M., Figuerêdo de Siqueira F. S. (2021). Effect of Addition of Dimethyl Sulfoxide to Simplified Adhesives on Dentin Bond Durability After 3 Years of Water Storage. *Journal of Adhesive Dentistry*.

[20] Burrer P., Dang H., Par M., Attin T., Tauböck T. T. (2020). Effect of Over-Etching and Prolonged Application Time of a Universal Adhesive on Dentin Bond Strength. *Polymers*.

[21] Sato T., Takagaki T., Hatayama T., Nikaido T., Tagami J. (2021). Update on Enamel Bonding Strategies. *Frontiers in Dental Medicine*.

[22] Sebold M., André C. B., Carvalho R. M., Giannini M. (2019). Dry-Bonding to Dentin Using Alternative Conditioners Based on Iron-Containing Solutions or Nitric Acid. *Journal of the Mechanical Behavior of Biomedical Materials*.

[23] Stape T. H. S., Tjäderhane L., Abuna G., Sinhoreti M. A. C., Martins L. R. M., Tezvergil-Mutluay A. (2018). Optimization of the Etch-and-Rinse Technique: New Perspectives to Improve Resin–Dentin Bonding and Hybrid Layer Integrity by Reducing Residual Water Using Dimethyl Sulfoxide Pretreatments. *Dental Materials*.

[24] Betancourt D., Baldion P., Castellanos J. (2019). Resin-Dentin Bonding Interface: Mechanisms of Degradation and Strategies for Stabilization of the Hybrid Layer. *International Journal of Biomaterials*.

[25] Stape T. H. S., Seseogullari-Dirihan R., Tjäderhane L., Abuna G., Martins L. R. M., Tezvergil-Mutluay A. (2018). A Novel Dry-Bonding Approach to Reduce Collagen Degradation and Optimize Resin-Dentin Interfaces. *Scientific Reports*.

[26] Kaynar Z. B., Kazak M., Donmez N., Dalkilic E. E. (2021). The Effect of Additional Chlorhexidine and/or Ethanol on the Bond Strength of Universal Adhesives. *Journal of Adhesion Science and Technology*.

[27] Ayar M. K. (2019). A Review of Ethanol Wet-Bonding: Principles and Techniques. *European Journal of Dentistry*.

[28] Souza M. Y., Andrade J. L., Caneppele T. M. F., Bresciani E. (2020). Assessment of Nanohardness, Elastic Modulus, and Nanoleakage of the Adhesive Interface Using the Ethanol-Wet-Bonding Technique. *International Journal of Adhesion and Adhesives*.

[29] Lindblad R., Lassila L., Vallittu P., Tjäderhane L. (2021). The Effect of Chlorhexidine and Dimethyl Sulfoxide on Long-Term Sealing Ability of Two Calcium Silicate Cements in Root Canal. *Dental Materials*.

[30] Zhang Z., Li K., Yang H., Yu J., Huang C. (2022). The Influence of Dimethyl Sulfoxide on Resin–Dentin Bonding: A Systematic Review. *International Journal of Adhesion and Adhesives*.

[31] Keshvad M. A., Hooshmand T., Behroozibakhsh M., Davaei S. (2019). Interfacial Fracture Toughness of Self-Adhesive and Conventional Flowable Composites to Dentin Using Different Dentin Pretreatments. *Journal of Investigative and Clinical Dentistry*.

[32] Reedy E. D., Stavig M. E. (2020). Interfacial Toughness: Dependence on Surface Roughness and Test Temperature. *International Journal of Fracture*.

[33] Pongprueksa P., De Munck J., Karunratanakul K. (2016). Dentin Bonding Testing Using a Mini-Interfacial Fracture Toughness Approach. *Journal of Dental Research*.

[34] de Mello R. M. M., Alcântara B. A. R., França F. M. G., do Amaral F. L. B., Basting R. T. (2022). Dimethyl Sulfoxide Dentin Pretreatments Do Not Improve Bonding of a Universal Adhesive in Etch-and-Rinse or Self-Etch Modes. *The journal of adhesive dentistry*.

[35] Moharam L. M., Afifi R. H. (2023). Influence of Adhesive Application Method and Thermocycling on the Bonding Performance of Different Adhesive Systems to Dentin. *Journal of International Oral Health*.

[36] Hu B., Hu Y., Li X. (2022). Shear Bond Strength of Different Bonding Agents to Orthodontic Metal Bracket and Zirconia. *Dental Materials Journal*.

[37] Hakimaneh S. M. R., Shayegh S. S., Ghavami-Lahiji M., Chokr A., Moraditalab A. (2020). Effect of Silane Heat Treatment by Laser on the Bond Strength of a Repair Composite to Feldspathic Porcelain. *Journal of Prosthodontics*.

[38] Kazemi-Yazdi H., Saeed-Nezhad M., Rezaei S. (2020). Effect of Chlorhexidine on Durability of Two Self-Etch Adhesive Systems. *Journal of Clinical and Experimental Dentistry*.

[39] Borges A. L. S., Dal Piva A. M. de O., Moecke S. E., de Morais R. C., Tribst J. P. M. (2021). Polymerization Shrinkage, Hygroscopic Expansion, Elastic Modulus and Degree of Conversion of Different Composites for Dental Application. *Journal of Composites Science*.

[40] Yi L., Yu J., Han L., Li T., Yang H., Huang C. (2019). Combination of Baicalein and Ethanol-Wet-Bonding Improves Dentin Bonding Durability. *Journal of Dentistry*.

[41] Kuhn E., Farhat P., Teitelbaum A. P. (2015). Ethanol-Wet Bonding Technique: Clinical Versus Laboratory Findings. *Dental Materials*.

[42] Souza M. Y. de, Jurema A. L. B., Caneppele T. M. F., Bresciani E. (2019). 6-Month Performance of Restorations Produced With the Ethanol-Wet-Bonding Technique: A Randomized Trial. *Brazilian Oral Research*.

[43] Lenzi T. L., Soares F. Z. M., de Oliveira Rocha R. (2017). Does Bonding Approach Influence the Bond Strength of Universal Adhesive to Dentin of Primary Teeth?. *Journal of Clinical Pediatric Dentistry*.

[44] Mehmood N., Nagpal R., Singh U. P., Agarwal M. (2021). Effect of Dentin Biomodification Techniques on the Stability of the Bonded Interface. *Journal of Conservative Dentistry*.

[45] Guo J., Lei W., Yang H., Zhang Y., Zhao S., Huang C. (2017). Dimethyl Sulfoxide Wet-Bonding Technique May Improve the Quality of Dentin Bonding. *Journal of Adhesive Dentistry*.

[46] Al-Ani A. A. S., Mutluay M., Stape T. H. S., Tjäderhane L., Tezvergil-Mutluay A. (2018). Effect of Various Dimethyl Sulfoxide Concentrations on the Durability of Dentin Bonding and Hybrid Layer Quality. *Dental Materials Journal*.

[47] Cardenas A. F. M., Araujo L. C. R., Szesz A. L. (2021). Influence of Application of Dimethyl Sulfoxide on the Bonding Properties to Eroded Dentin. *The Journal of Adhesive Dentistry*.

[48] Sharafeddin F., Salehi R., Feizi N. (2016). Effect of Dimethyl Sulfoxide on Bond Strength of a Self-Etch Primer and an Etch and Rinse Adhesive to Surface and Deep Dentin. *Journal of Dentistry*.

[49] Becker T. D., Agee K. A., Joyce A. P. (2007). Infiltration/Evaporation-Induced Shrinkage of Demineralized Dentin by Solvated Model Adhesives. *Journal of Biomedical Materials Research Part B: Applied Biomaterials*.

[50] Moharamzadeh K., Hooshmand T., Keshvad A., Van Noort R. (2008). Fracture Toughness of a Ceramic–Resin Interface. *Dental Materials*.

[51] Tantbirojn D., Cheng Y.-S., Versluis A., Hodges J. S., Douglas W. (2000). Nominal Shear or Fracture Mechanics in the Assessment of Composite-Dentin Adhesion?. *Journal of Dental Research*.

